# Adrenaline nasal spray in emergency management: An initial expert opinion 

**DOI:** 10.5414/ALX02590E

**Published:** 2025-09-04

**Authors:** Regina Treudler, Knut Brockow, Kirsten Beyer, Ludger Klimek, Lars Lange, Sabine Schnadt, Johannes Ring, Margitta Worm

**Affiliations:** 1 Institute of Allergology, Charité – Universitätsmedizin Berlin, Corporate Member of Freie Universität Berlin and Humboldt-Universität zu Berlin, Berlin,; 2 Department of Dermatology and Allergology am Biederstein, Faculty of Medicine and Health, Technical University of Munich, Munich,; 3 Department of Pediatric Respiratory Medicine, Immunology and Critical Care Medicine, Charité Universitätsmedizin Berlin,; 4 German Center for Child and Adolescent Health (DZKJ), Berlin,; 5 Center for Rhinology and Allergology, Wiesbaden,; 6 Department of Pediatrics and Adolescent Medicine, St. Marien-Hospital, Bonn,; 7 German Allergy and Asthma Association (DAAB). (DAAB), Mönchengladbach, and; 8 Department of Dermatology, Venerology and Allergology, Charité – Universitätsmedizin Berlin, Berlin, Germany

**Keywords:** adrenaline, anaphylaxis, allergy, epinephrine, intranasal, nasal spray, expert opinion

## Abstract

Abstract. Adrenaline is the drug of choice for the treatment of anaphylaxis. Up to now, intramuscular administration using an autoinjector has been recommended in national and international guidelines as the first-line treatment for anaphylaxis. Various adrenaline autoinjectors are available on the German market as emergency medication for immediate treatment by medical laypersons and specialists. Recently, a nasally administered adrenaline preparation was introduced for the first time and is available on the market. There are mainly data on healthy control subjects, which show a good adrenaline level and an expected effect on blood pressure and heart rate. To date, there is little clinical experience in the world literature for patients with anaphylaxis in children/adolescents and none in adults or from Germany. Therefore, we would like to discuss theoretically the use of adrenaline via the nasal route of administration in the care of anaphylaxis patients and compare it with the intramuscular administration of adrenaline autoinjectors.

## Introduction 

Anaphylaxis is an acute, potentially life-threatening reaction that can affect the entire body and requires rapid and effective treatment [3, 10, 14]. The most common triggers of anaphylaxis are insect venoms, foods, and medications [14, 20], but many other, sometimes rare triggers have also been described [18, 19]. Intramuscular administration into the lateral thigh is established worldwide and is the first-line treatment for a systemic allergic reaction as soon as two or more organ systems are affected simultaneously or consecutively, but at the latest when manifestations extend beyond the skin and gastrointestinal tract and occur in the lower respiratory tract or cardiovascular system [14, 21]. However, there are various barriers to the quick and easy use of adrenaline via autoinjectors (AAI), such as administration by laypeople (including caregivers), in children, or in situations where there is uncertainty about the necessity of administration [16, 23][Table Table1]. In addition, AAI are not available during an emergency situation if they are not regularly carried by those affected [23]. According to data from the literature, pharmaceutical and medical personnel are also uncertain about when and how adrenaline should be administered intramuscularly in cases of anaphylaxis [7, 22]. Several products are available in Germany for the intramuscular administration of adrenaline: Fastjekt (Viatris, Troisdorf, Germany), Jext (ALK-Abello, Reinbek, Germany), Anapen (Bioprojet, Berlin, Germany). In recent years, nasal administration of adrenaline has been successfully developed as an alternative emergency treatment for anaphylaxis [2, 5, 12]. An intranasal adrenaline preparation (EURNeffy, ALK-Abello, Wedel, Germany [1]) was recently approved in Europe and has been available on the German market since June 26, 2025. In the following, we would like to discuss the importance of a nasal form of adrenaline administration for the treatment of anaphylaxis patients. 

## Adrenaline nasal spray 

Adrenaline nasal spray was developed for the emergency treatment of anaphylaxis. It is a spray that delivers a dose of 2 mg of adrenaline to the nasal mucosa. It is suitable for people weighing 30 kg or more. The product has already been approved in the USA, where also a dose variant containing 1 mg of adrenaline to be used for people aged 4 years and older and weighing between 15 kg and 30 kg is already available [2, 9]. Approval of the product containing 1 mg of adrenaline is also being sought in Europe. EURNeffy contains sodium metabisulfite and benzalkonium chloride as additives. Nasal administration of drugs is well established and has been introduced for the administration of a variety of other drugs [5], including diazepam and sumatriptan. The addition of a carrier substance (dodecyl maltoside, Intravail) facilitates the absorption of adrenaline by increasing the permeability of the mucosal cells [13]. This improves the absorption of adrenaline into the systemic circulation. 

## Handling 

The nasal adrenaline preparation is used topically as a nasal spray and does not require an injection. It is important not to test the spray with a test dose prior to use, as only one single dose can be administered. If there is no improvement in symptoms within the first 10 minutes, a second dose is recommended. The preparation is only available as a double pack. The shelf life is specified as 30 months from the date of manufacture. No special storage conditions are required, as the medication is designed to remain stable even at high temperatures. Training devices are available from the company for patient training. The currently available training devices are for single use only and are intended to raise awareness during training that no control spray should be administered. A reusable training device would be desirable so that the patient’s environment can also become more familiar with its use. 

## Effect and efficacy 

Studies in healthy subjects have shown that the nasal adrenaline preparation reaches serum levels comparable to those achieved by manual intramuscular administration of adrenaline using a syringe within a few minutes of application, and serum levels comparable to those achieved with Fastjekt after 15 minutes (Table 2). Comparable desired adrenergic effects are also achieved [2, 5, 8, 12]. The desired adrenergic effects were also demonstrated in cases of swelling of the nasal mucosa due to allergic rhinitis (shown in subjects after nasal provocation testing) and in patients with upper respiratory tract infections [11]. A few data on clinical use in children with immediate-type reactions after oral food provocation are available from a registration study in Japan and a case series in the USA [4]. In addition, data from an animal model (dogs) show that even in cases of hypotension, there is no effect on the absorption of nasal adrenaline in anesthetized dogs [17]. 

## Safety 

The most common side effect of nasal adrenaline administration is local irritation of the nasal mucosa. In addition, particularly after administration of a second dose, some subjects also experienced headaches, nervousness, and tremors [1, 2, 5, 12]. 

## Indication 

The indication for prescribing an adrenaline preparation is based on the recommendations of the guideline for acute treatment and management of anaphylaxis [8]. The choice of adrenaline preparation and form of administration (intramuscular vs. intranasal) should be based on shared decision making with the patient. Possible criteria to aid decision-making are listed in Table 3. 

## Expert opinion 

Based on pharmacokinetic and pharmacodynamic data in human subjects, intranasal administration of adrenaline using a nasal spray for the treatment or prevention of anaphylaxis has been approved as an emergency medication for the treatment of anaphylaxis in many countries. The possibility of nasal administration of adrenaline represents an enrichment of the therapeutic spectrum in the treatment of anaphylaxis. Which patients are suitable for intramuscular and which for nasal administration should currently be considered on an individual basis [6]. Data will be needed in the future to identify patient groups that particularly benefit from this route of administration. Health economic considerations should not be ignored [15]. Here, it can be taken into account whether adrenaline has already been used repeatedly (e.g., for food allergies) or whether it is a seasonal emergency medication (e.g., for insect venom allergies) that is unlikely to be needed for months at a time. In addition, it would be desirable to be able to prescribe a single adrenaline nasal spray. Although up to 10% of anaphylaxis patients require a second dose of adrenaline, the medication is often not used for years for various reasons and is discarded unused [7]. 

The experts were unable to reach a consensus on the question of prescribing an adrenaline auto-injector and an adrenaline nasal spray at the same time. The majority opinion was that, not least for economic reasons, a medical decision should be made regarding one or the other method of administration. On the other hand, few experts argued that the possibility of simultaneous prescription should be considered in selected cases. 

Overall, we hope that the barriers to adrenaline use – as they exist today for patients and other users – can be reduced with the introduction of the new preparation and that anaphylaxis will be treated more frequently than before with adrenaline as early as possible in accordance with guidelines. 

## Authors’ contributions 

Conceptualization and writing original draft: RT: Writing – review and editing: RT, KBr, KBe, LK, SSchn, JR, MW. 

## Funding 

No funding. 

## Conflict of interest 

Regina Treudler received fees for lectures and/or advisory boards from AbbVie, ALK-Abello, Almirall, Galderma, Mylan/Viatris, Leo Pharma, Novartis, Pfizer, and Sanofi. 

Knut Brockow received fees for lectures from ALK-Abello, Novartis, ThermoFisher, UpToDate Inc., Blueprint, HAL, Bencard, advisory boards from ThermoFisher, BioMarin Pharmaceutical Inc., AstraZeneca, Blueprint, Celldex, and material costs for research from ThermoFisher, Bühlmann, and Macro Array Diagnostics. 

Kirsten Beyer received fees for advisory boards or lectures from Aimmune Therapeutics, ALK-Abelló, Danone, HAL, Infectopharm, Kantar Health, Mylan/Viatris, Nestlé, Novartis, Stallergenes Greer. 

(Ludger Klimek received research grants from Allergy Therapeutics/Bencard, UK/Germany, ALK-Abelló, Denmark, Allergopharma, Germany; Aimmune, USA; ASIT Biotech, Belgium; AstraZeneca, Sweden, Bionorica, Germany; BioNTech, Germany, Biomay, Austria, Blueprint, USA, Boehringer Ingelheim, Germany, Celltrion, South Korea, Circassia, USA; Chiesi, Italy; Cytos, Switzerland; Curalogic, Denmark; HAL, Netherlands; Lofarma, Italy; Menarini, Italy; Viatris/Mylan, USA; Novartis, Switzerland, Leti, Spain; ROXALL, Germany; GlaxoSmithKline (GSK), United Kingdom; Sanofi, France; Stallergenes, France; Thermofisher, USA and/or has worked as a speaker or consultant for the above-mentioned pharmaceutical companies. Ludger Klimek is currently President of the Ärzteverband Deutscher Allergologen (AeDA), member of the Governance Committee and member of the Executive Board of the ENT Section of the European Academy of Allergology and Clinical Immunology (EAACI), Vice President of the German Academy for Allergy and Environmental Medicine, and Editor-in-Chief of AllergoJournal and AllergoJournal International. 

Lars Lange received fees for consulting and lectures from Infectopharm, DBV Technologies, Sanofi, HAL Allergy, Thermofisher Scientific, Nestle Nutritional Institute, Nutricia, Novartis. 

Johannes Ring received fees for consulting and lectures from AbbVie, ALLERGIKA, ALK, Pfizer, ThermoFisher, Sanofi, Viatris. Sabine Schnadt received fees for lectures from Aimmune Therapeutics, ALK-Albello, DBV Technologies, Mylan/Viatris, Stallergenes Greer. 

Margitta Worm reports honoraria and/or consulting fees from AbbVie, Aimmune Therapeutics, ALK-Abelló, Allergopharma, Almirall, Amgen, Biotest, Boehringer Ingelheim, DBV Technologies, Genzyme, Kymab, LEO Pharma, Eli Lilly, Mylan/Viatris, Novartis, Pfizer, Regeneron Pharmaceuticals Inc., Sanofi, Stallergenes Greer 

**Table 1. Table1:** Possible barriers that may lead to the non-use or non-carrying of an adrenaline autoinjector (AAI).

**Barriers to use**
Lack of knowledge among medical/pharmaceutical staff about guideline-compliant AAI use [7, 22]
Lack of knowledge among patients regarding use with regard to – possible dangers of anaphylaxis without adrenaline administration – timing and – practical procedure for AAI use [16, 23]
Refusal or delay in use due to administration via a needle or the recommendation to go to a clinic for monitoring after use
Fear of side effects
**Barriers to carrying the AAI**
Size/weight of the AAI Possible stigmatization
Temperature-dependent storage of the AAI

**Table 2.** Comparison: nasal vs. intramuscular administration with a single dose [1].

**Administration**	**Intramuscular ** **(auto-injector)**	**Intramuscular ** **(syringe)**	**Nasal**
Application	Previous standard	Alternative standard for medical personnel	New
Suitable for laypeople	Yes	No	Yes
Injection	Yes	Yes	No
Time (minutes) to maximum serum level (median/range)	10 (2 – 45)	45 (3.9 – 360)	20 – 30 (2 – 240)
Max. serum level in pg/mL* (mean)	581	277	448 – 485

*The Food and Drug Administration (USA) considered 175 pg/mL to be the lower limit for efficacy (communications from ALK-Abelló, Wedel, Germany, and ARS Pharmaceuticals, San Diego, CA, USA).

**Table 3.** Criteria to aid decision-making with regard to intramuscular or nasal administration of adrenaline by laypersons.

Questions		Preference	
		Intra-muscular	Nasal
1.	Has successful anaphylaxis patient training already been carried out with an AAI?	++	+
2.	Has training been provided to caregivers using AAI?	++	+
3.	Has an AAI been used correctly by the patient before?	++	(+)
4.	Is the body weight less than 30 kg?	+++	–
5.	Are there any difficulties with the application technique of the AAI (e.g., visual impairment)?	–	+++
6.	Was the AAI not carried in the past due to size, storage, or portability issues?	+	++
7.	Has the AAI not been used in the past despite an acute reaction, e.g., due to reservations about an injection at the daycare center?	(+)	+++
8.	Is there such a thing as needle phobia?	–	+++
9.	Is there a reluctance to use new preparations?	++	(+)
10.	Are high or low storage temperatures to be expected (e.g., outdoor sports, travel to countries with high temperatures)?	(+)	+++
11.	Are there any pre-existing conditions of the nose that may preclude nasal administration of adrenaline (e.g., previous surgery, empty nose syndrome)?	+++	–
12.	Is there a contact allergy to benzalkonium chloride?	+++	(+)
13.	Is there a preference for a particular application?	+++	+++
14.	Is adrenaline mainly needed seasonally, making a longer shelf life advantageous?	+	++

Degree of preference: +++ very strong, ++ strong, + less strong, (+) possible, – not suitable; AAI = adrenaline auto-injector.
